# A novel method to calculate compliance and airway resistance in ventilated patients

**DOI:** 10.1186/s40635-022-00483-2

**Published:** 2022-12-30

**Authors:** Guillermo Gutierrez

**Affiliations:** Professor Emeritus Medicine, Anesthesiology and Engineering, The George Washington University, 700 New Hampshire Ave, NW, Suite 510, Washington, DC 20037 USA

**Keywords:** Mechanical ventilation, Acute respiratory failure, Static compliance, Airway resistance, Numerical analysis, Frequency analysis

## Abstract

**Background:**

The respiratory system’s static compliance (*C*_rs_) and airway resistance (*R*_rs_) are measured during an end-inspiratory hold on volume-controlled ventilation (static method). A numerical algorithm is presented to calculate *C*_rs_ and *R*_rs_ during volume-controlled ventilation on a breath-by-breath basis not requiring an end-inspiratory hold (dynamic method).

**Methods:**

The dynamic method combines a numerical solution of the equation of motion of the respiratory system with frequency analysis of airway signals. The method was validated experimentally with a one-liter test lung using 300 mL and 400 mL tidal volumes. It also was validated clinically using airway signals sampled at 32.25 Hz stored in a historical database as 131.1-s-long epochs. There were 15 patients in the database having epochs on volume-controlled ventilation with breaths displaying end-inspiratory holds. This allowed for the reliable calculation of paired *C*_rs_ and *R*_rs_ values using both static and dynamic methods. Epoch mean values for *C*_rs_ and *R*_rs_ were assessed by both methods and compared in aggregate form and individually for each patient in the study with Pearson’s *R*^2^ and Bland–Altman analysis. Figures are shown as median[IQR].

**Results:**

Experimental method differences in 880 simulated breaths were 0.3[0.2,0.4] mL·cmH_2_O^−1^ for *C*_rs_ and 0[− 0.2,0.2] cmH_2_O·s· L^−1^ for *R*_rs_. Clinical testing included 78,371 breaths found in 3174 epochs meeting criteria with 24[21,30] breaths per epoch. For the aggregate data, Pearson’s *R*^2^ were 0.99 and 0.94 for *C*_rs_ and *R*_rs_, respectively. Bias ± 95% limits of agreement (LOA) were 0.2 ± 1.6 mL·cmH_2_O^−1^ for *C*_rs_ and − 0.2 ± 1.5 cmH_2_O·s· L^−1^ for *R*_rs_. Bias ± LOA median values for individual patients were 0.6[− 0.2, 1.4] ± 0.9[0.8, 1.2] mL·cmH_2_O^−1^ for *C*_rs_ and − 0.1[− 0.3, 0.2] ± 0.8[0.5, 1.2] cmH_2_O·s· L^−1^ for *R*_rs_.

**Discussion:**

Experimental and clinical testing produced equivalent paired measurements of *C*_rs_ and *R*_rs_ by the dynamic and static methods under the conditions tested.

**Conclusions:**

These findings support to the possibility of using the dynamic method in continuously monitoring respiratory system mechanics in patients on ventilatory support with volume-controlled ventilation.

**Supplementary Information:**

The online version contains supplementary material available at 10.1186/s40635-022-00483-2.

The respiratory system (rs) static compliance (*C*_rs_) and airway resistance (*R*_rs_) are calculated during volume-controlled (VC) mechanical ventilation with a breath-hold maneuver at the end of quiet inspiration (static method) [1]. Under these conditions *C*_rs_ = *V*_tidal_ /(*P*_plateau_ – PEEP_a_), where *V*_tidal_ = tidal volume, *P*_plateau_ = breath-hold *P*_aw_, and PEEP_a_ = applied positive end expiratory pressure. Similarly, *R*_rs_ = (*P*_peak_ – *P*_plateau_)/*F*_aw_, where *P*_peak_ = peak inspiratory pressure and F_aw_ is airway flow measured just prior to breath-holding [2].

A reliable method to calculate *C*_rs_ and *R*_rs_ automatically, without the need of an inspiratory hold, would have great utility in monitoring the adequacy of ventilatory support. One approach previously tried is the multiple least squares fit (LSF) technique [3, 4], where measures of *P*_aw_, *F*_aw_, and lung volume change (Δ*V*) are fitted to the equation of motion of the respiratory system. Another is the expiratory time constant τe method [5] where equations for *C*_rs_ and *R*_rs_ are developed assuming mono-exponential lung volume release [6]. Both methods require the use of complex computational techniques and absent respiratory muscle effort.

Described is a method to calculate *C*_rs_ and *R*_rs_ during insufflation in the presence of airflow (dynamic method) that combines frequency analysis of the airway signals with a novel numerical solution of the equation of motion. The method was validated experimentally with a one-liter test lung. It was also validated clinically using previously acquired *F*_aw_ and *P*_aw_ signal data from patients on VC ventilation displaying end-inspiratory holds. This allowed for the reliable calculation of paired *C*_rs_ and *R*_rs_ values using both static and dynamic methods.

*Theoretical development*. The time-dependent equation of motion of the respiratory system is:

1 $${{{{P}_{\mathrm{aw}}\left(t\right)=P}_{\mathrm{mus}}\left(t\right)+ P}_{\mathrm{vent}}\left(t\right)=\frac{\Delta V\left(t\right)}{{C}_{\mathrm{rs}}}+ {R}_{\mathrm{rs}}{F}_{\mathrm{aw}}\left(t\right)+ I\frac{{\mathrm{d}}^{2}V\left(t\right)}{\mathrm{d}{t}^{2}}+ {\mathrm{PEEP}}_{a}+ {\mathrm{PEEP}}_{i}}$$

This equation, based on the one-compartment model of Otis et al. [7], assumes constant values for *C*_rs_ and *R*_rs_. The measured airway pressure *P*_aw_(*t*) represents the sum of the ventilator and respiratory muscles applied pressures *P*_vent_(*t*) and *P*_mus_(*t*), respectively. Opposing them are the elastic, resistive, and inertial components of the respiratory system. *V*(*t*) represents the time-dependent lung volume; Δ*V*(*t*) is the insufflation lung volume at time *t*, equal to ${\int }_{0}^{t}{F}_{aw}\left(t\right)dt$; I is the respiratory system inertia; and PEEP_i_ the intrinsic PEEP [8].

Assuming passive insufflation (*P*_mus_ = 0), negligible PEEP_I,_ and ignoring the effect of the inertia term [9]_,_ Eq. (1) becomes:

2 $${P}_{\mathrm{aw}}\left(t\right)= {P}_{\mathrm{vent}}\left(t\right)= \frac{\Delta V\left(t\right)}{{C}_{\mathrm{rs}}}+ {R}_{\mathrm{rs}} {F}_{\mathrm{aw}}\left(t\right)+ {\mathrm{PEEP}}_{a}.$$

It is possible to solve numerically this indeterminate equation with two unknowns, *C*_rs_ and *R*_rs_, by first developing a solution matrix for each set of *P*_aw_(*t*_k_), Δ*V*(*t*_k_), *F*_aw_(*t*_k_), and PEEP_a_ values measured at successive times *t*_k_ during insufflation. The elements of the solution matrix are calculated by substituting the measured values for Δ*V*(*t*_k_), *F*_aw_(*t*_k_), and PEEP_a_ into Eq. 2 and alternately applying a range of physiologically plausible values for *C*_rs_ (*C*_1_ … *C*_*n*_) and *R*_rs_ (*R*_1_ … *R*_*n*_).

$$\begin{array}{ccc}\ \ \ \ \ \ \ \ \ \ \ \ \ \ \ \ \ \ \ \ \ \ \ \ \ \ {R}_{1}\ \ \ \ \ \ \ \ \ \ \ \ \ \ \ \ & {R}_{2}\ \ \ \ \ \ \ \ \ \ \ \ \ \ \ \ \ \ \ \ \ \ \ \ \ & {R}_{n} \end{array}$$

$${\mathrm{Solution}}\ {\mathrm{matrix}}=\left[ \begin{array}{*{20}c}{P}_{\mathrm{aw} }\left({R}_{1},{C}_{1}\right)& {P}_{\mathrm{aw} }({R}_{2},{C}_{1})& \cdots & {P}_{\mathrm{aw} }\left({R}_{n}{,C}_{1}\right)\\ \vdots & \vdots & & \vdots \\ {P}_{\mathrm{aw}}\left({R}_{1},{C}_{n}\right)& {P}_{\mathrm{aw}}({R}_{2},{C}_{n})& \cdots & {P}_{\mathrm{aw}}\left({R}_{n},{C}_{n}\right)\end{array}\right] \begin{array}{*{20}c}{C}_{1}\\ \vdots \\ {C}_{n}\end{array}$$

For example, applying a range of (*C*_1_ … *C*_*n*_) values from 10 to 100 mL·cmH_2_O ^−1^ and 1.0 to 50.0 cmH_2_O·s·L^−1^ for (*R*_1_ … *R*_*n*_), at intervals of 0.1 each, produces a 900 × 490 solution matrix containing all possible *P*_aw_ values capable of satisfying Eq. 2 for given a set of (*t*_k_), *F*_aw_(*t*_k_), and PEEP_a_ measurements made at time *t*_k_ during insufflation.

Figure 1 shows a schematic of the proposed numerical method of solution. In this example, a solution matrix was generated for Δ*V*(*t*_k_) = 300 mL, *F*_aw_(*t*_k_) = 32 L·min^−1^, and PEEP_a_ = 5 cmH_2_O and plotted as a three-dimensional surface in a Cartesian (*C*_rs_, *R*_rs_, *P*_aw_) system. According to the above reasoning, the solution of Eq. 2, in terms of *C*_rs_ and *R*_rs_, resides on a point on that surface. Further insight is gained by noting that the solution must lie along a surface path traced by the measured *P*_aw_(*t*_k_) at time *t*_k_. This is shown in Fig. 1 as path A, where *P*_aw_(*t*_k_) = 27 cmH_2_O and point 'a' symbolizes the yet unknown solution of Eq. 2.

**Fig. 1:**
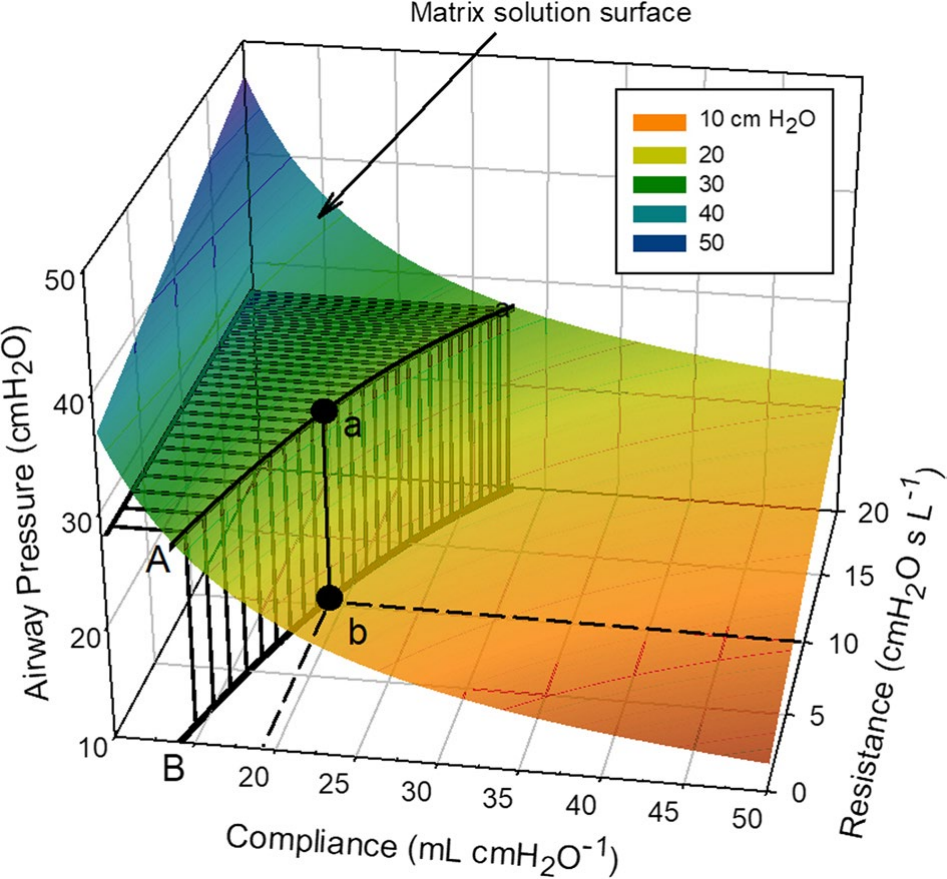
Schematic of the numerical method used to solve the respiratory system equation of motion for static compliance (*C*_rs_) and airway resistance (*R*_rs_). In this example, the solution matrix was developed for Δ*V*(*t*_k_) = 300 mL, *F*_aw_(*t*_k_) = 32 L·min^−1^, and PEEP_a_ = 5 cmH_2_O and shown graphically as a three-dimensional surface bounded by *C*_rs_ values ranging from 10 to 50 mL·cmH_2_O^−1^ and *R*_rs_ from 0 to 20 cmH_2_O·s·L^−1^. This surface encompasses all possible combinations of *P*_aw_, *C*_rs_ and *R*_rs_ capable of satisfying Eq. 2 for a given set of Δ*V*, *F*_aw_, and PEEP_a_ measurements made at time t_k_ during insufflation. *P*_aw_, also measured at t_k_ and equal in this example to 27 cmH_2_O, further restricts the solution of Eq. 2 to lie along path (**A**). This path is defined by surface values coinciding with the measured *P*_aw,_ with point ‘a’ referring to the still unknown solution of Eq. 2. Projecting path A onto the *C*_rs_ – *R*_rs_ plane results in a two-dimensional function (**B**) relating *C*_rs_ to *R*_rs_. Here ‘b’ represents the unique solution of Eq. 2 defining the values for *C*_rs_ and *R*_rs_ for the breath under consideration

Projecting path (A) onto the *C*_rs_–*R*_rs_ plane generates a two-dimensional function, shown as path B, that restricts all possible combinations of *C*_rs_ and *R*_rs_ able to satisfy Eq. 2 for the set of measurements taken at time *t*_k_. The *C*_rs_–*R*_rs_ function is developed numerically by noting the values *R*_x_, *C*_y_ associated with those matrix elements where *P*_aw_(*R*_x_, *C*_y_) = measured *P*_aw_(*t*_k_).

It only remains to identify the location of solution point ‘b’ on the *C*_rs_–*R*_rs_ plane. This is accomplished by generating a family of *C*_rs_–*R*_rs_ functions, one for each set of *P*_aw_(*t*_k_), Δ*V*(*t*_k_), *F*_aw_(*t*_k_), and PEEP_a_ values measured at sequential times t_k_ during insufflation. Since the one-compartment model of Eq. 2 assumes constant *C*_rs_ and *R*_rs_, it follows that all generated *C*_rs_–*R*_rs_ functions must pass through, and therefore intersect, at a point that defines *C*_rs_ and *R*_rs_ for the breath in question.

It is known that *C*_rs_ and *R*_rs_ vary early in inspiration as unstable alveoli open and conducting airways distend [10]. However, as lung volume increases past a lower inflection point (LIP) *C*_rs_ achieves steady state until reaching an upper inflection point (UIP) where over-distention might occur. Defining the LIP and UIP by their respective lung volumes as Δ*V*_LIP_ and Δ*V*_UIP_, it is reasonable to expect all *C*_rs_–*R*_rs_ functions generated for insufflation lung volumes Δ*V*_LIP_ < Δ*V*(*t*) < Δ*V*_UIP_ to intersect at the solution point ‘b’ uniquely defining *C*_rs_ and *R*_rs_ for that breath.

Figure 2 shows a family of *C*_rs_–*R*_rs_ functions (*n* = 14) generated during a single breath’s insufflation at sequential 32-ms intervals, past Δ*V*_LIP_ > 200 mL. The intersection of these functions defines the values for *C*_rs_ = 32.8 mL·cmH_2_O ^−1^ and *R*_rs_ = 23.8 cmH_2_O·s·L^−1^. The inset graph illustrates the slight uncertainty associated in determining the intersection of the *C*_rs_–*R*_rs_ functions, likely the result of random variations in measurement or small changes in *C*_rs_ and *R*_rs_ occurring during the insufflation. Accordingly, the point of intersection is best defined by the smallest standard deviation (*σ*) of all *C*_rs_ values measured at each *R*_rs_ increment along the *R*_rs_ axis.

**Fig. 2:**
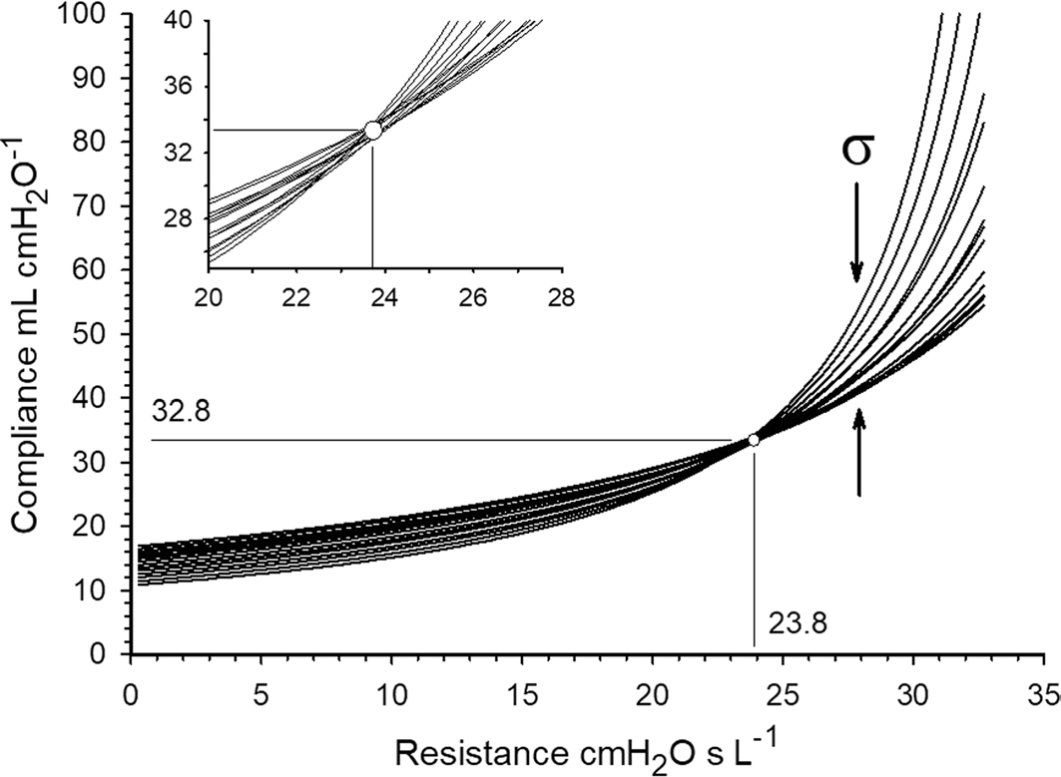
A family of *C*_rs_ and *R*_rs_ functions (*n* = 14). Each function was generated at different times (*t*_k_) measured sequentially at 32 ms during a single insufflation. The intersection of these functions defines *C*_rs_ = 32.8 mL·cmH_2_O ^−1^ and *R*_rs_ = 23.8 cmH_2_O·s· L^−1^ for the breath. Shown in the inset graph is the uncertainty associated with the intersection point, likely the result of measurement limitations or minute alterations in *C*_rs_ and *R*_rs_ during insufflation. Accordingly, the point of intersection is best defined by the smallest standard deviation (*σ*) of all *C*_rs_ values measured at each *R*_rs_ increment along the *R*_rs_ axis

## Methods

The accuracy of the dynamic method was tested by comparing paired *C*_rs_ and *R*_rs_ values predicted by the dynamic method and the static method (used here as the ‘gold standard’) for the same breath.

### Experimental validation

Validation was performed experimentally with a Maquet 190 one-liter test lung (Getinge, Solna, Sweden) using VC ventilation with a 0.5-s inspiratory hold. The test lung was attached to a Servo s ventilator (Getinge, Solna, Sweden) and ventilated at a respiratory rate of 15 bpm with *V*_tidal_ of 300 mL or 400 mL. PEEP levels of 0, 5 and 10 cmH_2_O were applied sequentially at each *V*_tidal_.

An in-house built data acquisition monitor was used to sample *F*_aw_ and P_aw_ signals from the ventilator data-port at 32.25 Hz and compile successive epochs of 4096 points, each lasting 131.1 s. Five epochs were obtained at each *V*_tidal_–PEEP combination. Data were analyzed in situ with the monitor’s Raspberry Pi 3B processor programmed (Python 3.8) to calculate *C*_rs_ and *R*_rs_ for each breath by the dynamic method. *C*_rs_ and *R*_rs_ were also determined manually by the static method for 10 breaths in each epoch using data from the *P*_aw_ and *F*_aw_ signals. Average epoch values for *C*_rs_ and *R*_rs_ computed with either method were compared at each *V*_tidal_–PEEP combination.

### Clinical validation

The dynamic method also was validated with clinical data using *F*_aw_ and *P*_aw_ signals obtained in a prior study of mechanically ventilated patients performed in 2011–2012 at The George Washington University Hospital Intensive Care Unit (IRB No. 110910) [11]. The database (Additional file 1: Section S1) contains information from 176 patients with acute respiratory failure enrolled within 24 h of intubation and monitored during their entire time on ventilatory support. It contains deidentified demographic information and *F*_aw_ and *P*_aw_ signals sampled at 32.25 Hz by the ventilator (Servo I or Servo S ventilators, Getinge, Solna, Sweden). The signals were saved as contiguous time-windows or epochs, each lasting 131.1 s and containing 4096 samples of each signal.

### Epoch selection

Software was written (Python 3.8) to search the database for epochs on VC ventilation. The respiratory rate variability (RRV) for each identified epoch was used to determine the degree of active respiratory muscle activity. RRV was determined from the frequency spectrum of the expiratory flow signal as previously described [12] using the fast Fourier transform (FFT) algorithm [13]. RRV was defined as 100 – H1/DC %, where H1 is the amplitude of the spectrum’s first harmonic and DC that of the zero-frequency component. Epochs with RRV < 55% were assumed to have negligible respiratory muscle activity (*P*_mus_ = 0) and were chosen for the study. This RRV value corresponds to those noted in normal individuals during quiet breathing in stages N2 and N3 of sleep [14].

### Breath selection

Within each selected epoch, the software further identified breaths displaying an end-inspiratory hold and absent voluntary respiratory effort. These breaths allowed for the reliable measurements of static compliance and airway resistance using standard calculations for comparison with those predicted by the dynamic method. The following criteria was used to choose breaths for analysis: (1) a discernible end-inspiratory hold > 0.25 s with mean plateau airway flow < 1 L·min^−1^; (2) ventilator-triggered (PEEP_a_ – minimal *P*_aw_ < 0.3 cmH_2_O); (3) full volume breaths (Δ*V*(*t*) ≥ 300 mL with insufflation time (Ti) > 0.8 s); (4) absent PEEP_i_ (end-exhalation (EE) *F*_aw_ < 3 L·min^−1^ [15] and *P*_aw_(t_0_)—EE *P*_aw_ < 2 cmH_2_O) [16]; and (5) no air circuit leak (inspired – expired *V*_tidal_ <|30 mL|). Excluded were breaths with Δ*V*(*t*) ≥ 740 mL to avoid exceeding the UIP [17] (see Additional file 1: Section S2 for breath exclusion example). Once a breath was deemed adequate for analysis, the software calculated *C*_rs_ and *R*_rs_ by both the dynamic and static methods.

Results from the dynamic and static methods were compared with Pearson’s linear regression *R*^2^ and Bland–Altman analysis [18] for bias ± 95% limits of agreement (LOA). Since some patients had substantially more epochs meeting study criteria than others, the methods were compared in aggregate by combining data from all epochs and individually for each study patient. Unless otherwise specified, data are shown as median and interquartile range. The Mann–Whitney test was used to determine significant differences between independent samples. All reported *p* values are two-sided with *p* < 0.05 considered significant.

## Results

### Experimental validation

Analysis of 880 breaths from 30 epochs resulted in nearly identical values for *C*_rs_ and *R*_rs_ calculated by the static and dynamic methods for all tested combinations of *V*_tidal_ and PEEP (Table 1; *p* = NS). Overall method differences were 0.3[0.2,0.4] mL·cmH_2_O ^−1^ for *C*_rs_ and 0[− 0.2,0.2] cmH_2_O·s·L^−1^ for *R*_rs_.

**Table 1:** Respiratory system’s static compliance and resistance values calculated by the static and dynamic methods using a test lung

*V*_tidal_	PEEP*	Compliance mL·cmH_2_O ^−1^		Resistance cmH_2_O·s·L^−1^	
mL	cmH_2_O	Static	Dynamic	Static	Dynamic
	0	21.0[21.0,21.0]	21.5[21.4,21.5]	7.9[7.9,8.0]	7.7[7.6,7.8]
300	5	22.8[22.8,22.9]	23.2[23.1,23.3]	8.2[5.2,8.3]	8.3[8.2,8.3]
	10	26.1[26.0,26.1]	26.4[26.4,26.3]	8.2[7.9,8.2]	8.1[8.1,8.3]
	0	22.9[22.9,23.0]	23.0[23.0,23.1]	10.5[10.4,10.5]	10.1[10.0,10.1]
400	5	24.4[24.4,24.5]	24.7[24.6,24.8]	10.7[10.7,10.8]	11.0[11.0,11.0]
	10	27.7[27.7,27.8]	28.1[28.0,28.1]	10.7[10.7,11.1]	11.0[10.9,11.0]
Total (n = 880)		23.6[22.8,26.1]	23.9[23.0,26.4]	9.3[8.2,10.7]	9.2[8.1,10.9]
Difference^†^		0.3 [0.2,0.4]		0 [− 0.2,0.2]	

There were no significant differences between methods in any of the variables measured

*V*_tidal_  tidal volume, *PEEP*  positive end expiratory pressure; values shown as median [IQR]

^*^Five epochs per PEEP level, each containing approximately 30 breaths

^**†**^Difference = dynamic – static methods

### Clinical validation

Of the 176 patients in the database, 15 (8.5%) were identified as meeting study criteria. The 15 patients had a combined total of 33,371 epochs on VC ventilation and RRV < 55%. The study patients were evenly split according to gender, but ranged widely in age, predicted body weight (PBW) and body mass index (BMI). Disease acuity was high (SAPS II 36[32,44]), five were non-cardiac post-operative, two were trauma and the remainder medical patients. The *P*/*F* ratio was relatively high at 337[272,429] mmH_2_O, reflecting the lack of lung pathology noted in half of the patients’ chest radiographs (Additional file 1: Table S1, Additional file 2, Additional file 3, Additional file 4).

Of the 33,371 identified epochs, 3174 (9.5%) contained breaths displaying end-inspiratory holds. The ventilatory parameters associated with these epochs were compatible with those of quiet, passive ventilation with a low RR = 11[11,14] bpm and RRV = 45 [40,46] % (Additional file 1: Table S2). The 3174 chosen epochs encompassed 87,021 individual breaths with 78,371 (90.1%) considered adequate for analysis of static compliance and airway resistance using standard calculations for comparison with those predicted by the dynamic method. The median number of breaths in these epochs was 24[21,30].

### Aggregate data analysis

**Fig. 3:**
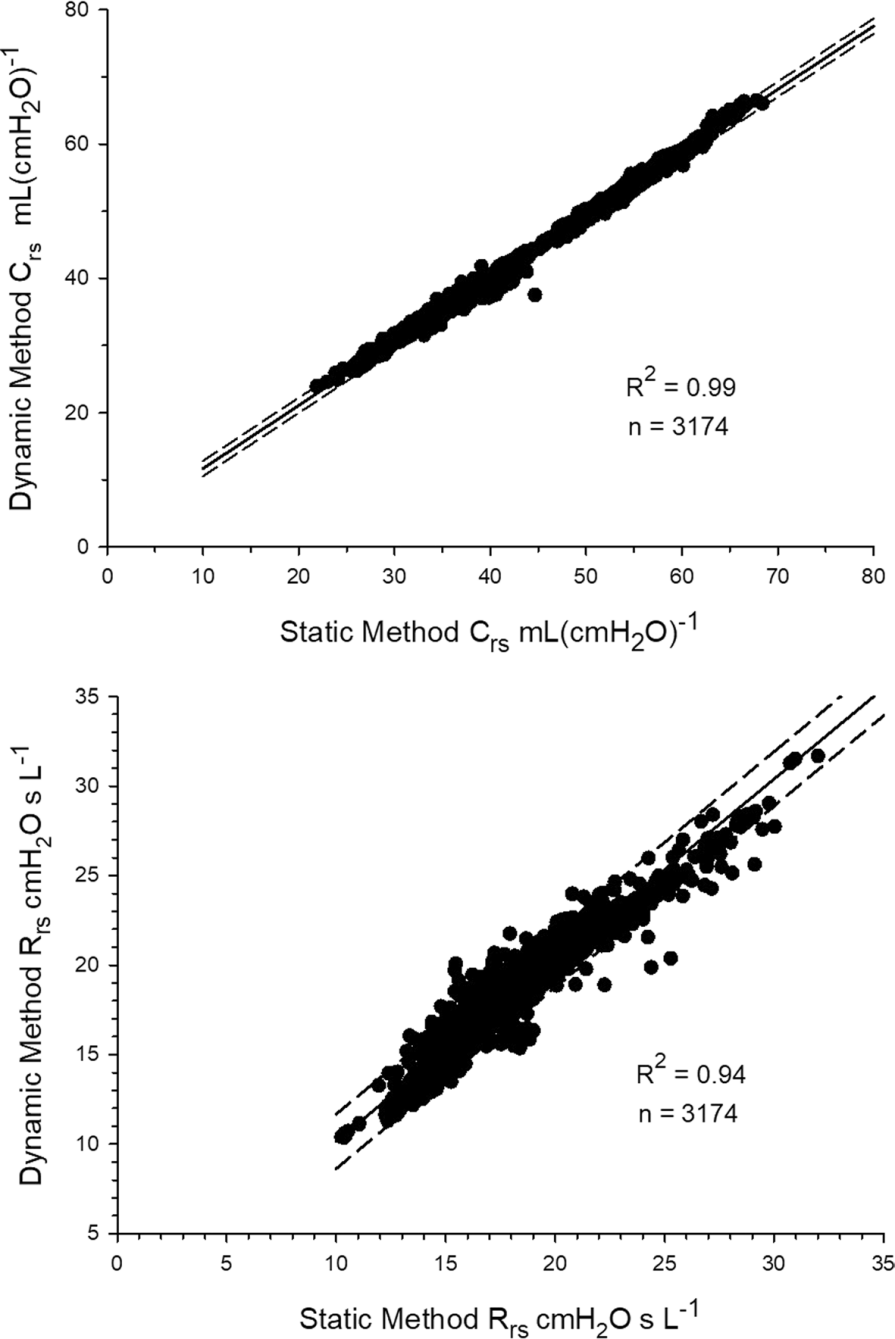
Pearson’s linear regression using data generated by the 15 patients in the study. Compared were average epoch measurements of *C*_rs_ and of *R*_rs_ by the static and dynamic methods (*n* = 3174). (*C*_rs_)_stat_ = 1.06 (*C*_rs_)_dyn_ – 2.26; *R*^2^ = 0.99; *p* < 0.001 and (*R*_rs_)_stat_ = 0.93 (*R*_rs_)_dyn_ + 1.02; *R*^2^ = 0.94; *p* < 0.001

**Fig. 4:**
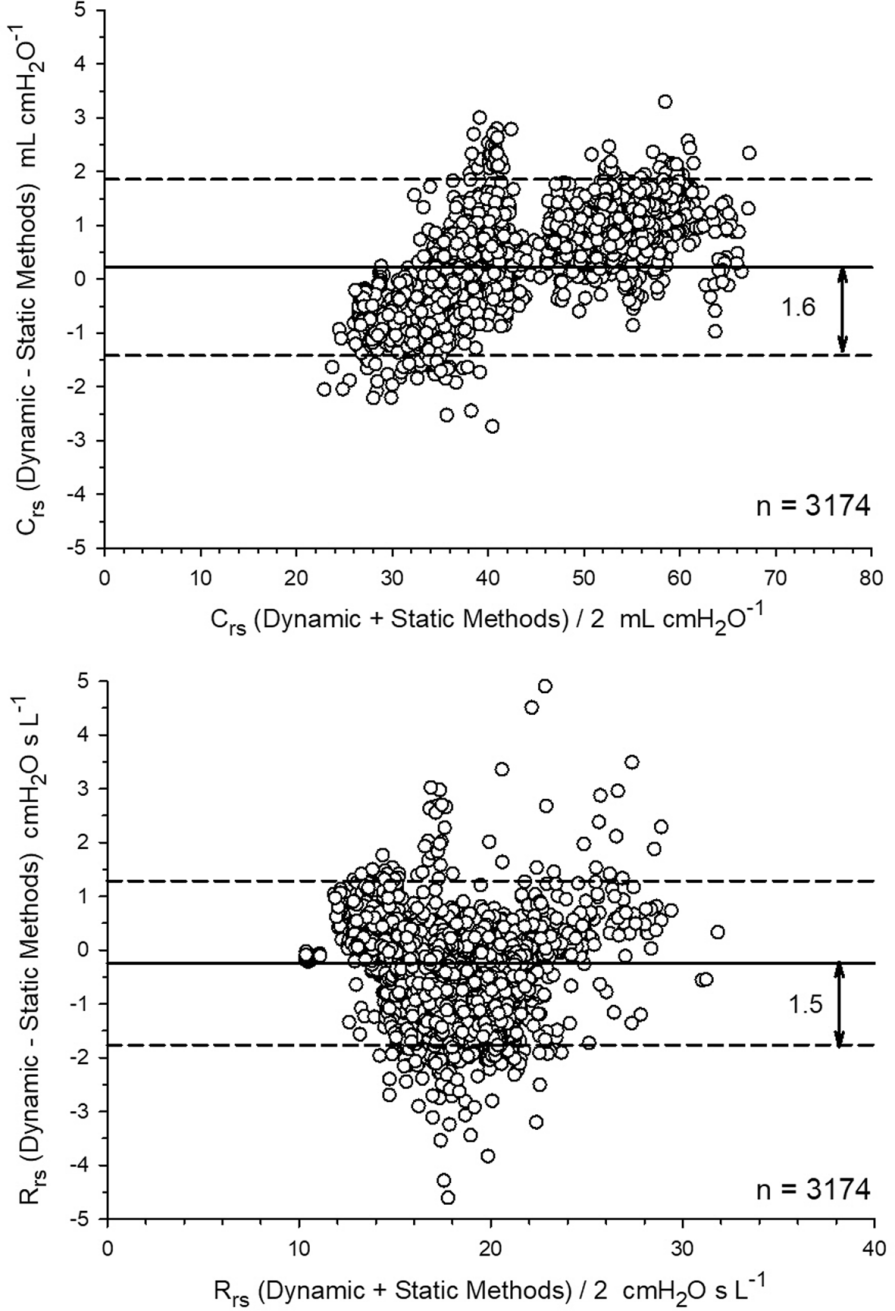
Bland–Altman analysis of average epoch measurements of *C*_rs_ and *R*_rs_ (*n* = 3174) from the data generated by the 15 patients in the study. Bias ± 95% LOA was 0.2 ± 1.6 mL·cmH_2_O ^−1^ for *C*_rs_ and − 0.2 ± 1.5 cmH_2_O·s· L^−1^ for *R*_rs_

There was an excellent correlation between the static and dynamic methods (Fig. 3) with (*C*_rs_)_stat_ = 1.06 (*C*_rs_)_dyn_ – 2.26; *R*^2^ = 0.99; *p* < 0.001 and (*R*_rs_)_stat_ = 0.93 (*R*_rs_)_dyn_ + 1.02; *R*^2^ = 0.94; *p* < 0.001. Bland–Altman analysis (Fig. 4) showed bias ± LOA of 0.2 ± 1.6 mL·cmH_2_O ^−1^ for *C*_rs_ and – .2 ± 1.5 cmH_2_O·s· L^−1^ for *R*_rs_.

*Individual patient analysis.* Table 2 shows Bland–Altman analyses for individual study patients. There were 119 [34, 339] (range 15 to 881) epochs per patient containing 2926 [624, 7702] (range 426 to 17,953) breaths. Individual patient bias ± LOA for *C*_rs_ was 0.6 [− 0.2, 1.4] (range − 0.8 to 1.6) ± 0.9 [0.8, 1.2] (range 0.7 to 2.3) mL·cmH_2_O ^−1^. Bias ± LOA for *R*_rs_ was − 0.1[− 0.3, 0.2] (range − 1.6 to 2.1) ± 0.8 [0.5, 1.2] (range 0.2 to 2.2) cmH_2_O·s· L^−1^.

**Table 2:** Bland–Altman analysis of respiratory system’s static compliance and resistance values calculated by the static and dynamic methods for individual patients

Patient	Epochs (3174)	Breaths (78,371)	Compliance mL·cmH_2_O ^−1^			Resistance cmH_2_O·s·L^−1^		
			Mean value	Bias	LOA	Mean value	Bias	LOA
1	293	5924	35.8	0.4	1.1	21.9	0.4	1.4
2	119	4917	27.5	− 0.6	0.9	15.2	0.2	2.2
3	127	2926	58.6	1.4	0.9	14.3	0.2	0.3
4	385	8632	32.8	− 0.3	1.2	20.5	− 0.3	0.9
5	15	459	59.7	1.6	0.9	16.7	− 0.1	0.6
6	38	660	46.7	1.6	2.3	13.6	− 0.4	1.9
7	399	11,714	39.7	0.2	0.9	15.4	0.1	1.1
8	881	17,953	53.5	0.8	0.8	16.8	− 0.3	0.6
9	72	1718	49.7	1.3	0.8	17.7	-0.1	0.5
10	27	426	41.2	1.4	1.3	17.4	2.1	1.1
11	38	1123	47.5	0.7	1.1	12.2	0.8	0.4
12	26	494	52.5	0.0	0.7	12.0	− 0.1	0.2
13	248	6771	34.8	− 0.8	0.7	18.9	− 1.6	0.8
14	476	14,067	33.8	− 0.5	1.2	15.6	− 0.5	1.6
15	30	587	64.7	0.6	1.3	12.9	0.2	0.3
Median	119	2926	46.7	0.6	0.9	15.6	− 0.1	0.8
IQR	[34,339]	[624,7702]	[35.3, 53.0]	[− 0.2, 1.4]	[0.8, 1.2]	[13.9, 17.5]	[− 0.3, 0.2]	[0.5, 1.2]

Mean value = average value of all measurements used in the Bland–Altman analysis; LOA = 95% limits of agreement; IQR = interquartile range]

## Discussion

Increases in computing power [19] allow for the application of powerful analytical techniques to monitor patients on ventilatory support. The present study describes an algorithm capable of providing breath-by-breath measures of *C*_rs_ and *R*_rs_ without the need for an end-inspiratory pause in patients on VC ventilation. This technique may in turn allow for the continuous monitoring of other parameters, such as the driving pressure, a proven indicator of ventilator associated lung injury [20].

The dynamic method used to determine static *C*_rs_ and *R*_rs_ is based on a novel numerical solution of the equation of motion of the respiratory system. This equation depicts the behavior of respiratory mechanics in normal individuals and has been applied successfully to ventilated patients with respiratory failure [21]. In the form used here, the equation of motion ignores the inspired gas inertia and the resistance to energy transfer by visco-elastic lung tissue, whereas both terms may be quantitatively significant under extreme ventilatory conditions, they are likely inconsequential under the studied conditions [22]. It should be noted that the one-compartment model of Otis et al. [7] does not allow for the partitioning of respiratory system mechanics. On the other hand, a similar numerical approach may be considered when solving a more complex model of the respiratory system, one that accounts for both lung and chest wall compliances.

Method validation was done with matching pairs of *C*_rs_ and *R*_rs_ calculated by the static and the dynamic methods. Experimental method validation yielded nearly identical *C*_rs_ and *R*_rs_ values when tested with a test lung ventilated using different *V*_tidal_ and applied PEEP levels. Since *C*_rs_ and *R*_rs_ were more or less fixed for the test lung, the dynamic method also was validated using airway signals from a previous study on mechanically ventilated patients. The use of clinical data yielded a more realistic assessment of the dynamic method, allowing for method comparison at *C*_rs_ values ranging from 20 to 70 mL·cmH_2_O^−1^ and from 10 to 32 cmH_2_O·s· L^−1^ for *R*_rs_. These are ranges similar to those encountered in clinical practice.

Software was written to identify breaths meeting strict morphologic criteria that included a discernible plateau pressure and negligible *P*_mus_ or PEEP_i_. This resulted in the evaluation of a massive number of individual breaths (78,371) contained in the 3174 identified epochs. The software calculated paired *C*_rs_ and *R*_rs_ values by the static and dynamic methods in all identified breaths enclosed within each 131.1-s-long epoch, reporting the epoch’s average for comparison. The use of epochs was dictated both by the format initially used to store the data and by the ability to assess respiratory muscle activity indirectly by spectral analysis.

The cohort was composed mainly of highly sedated patients transferred from the Emergency Department and ventilated with end-inspiratory holds that were not immediately detected by the ICU team. Although the data were collected several years ago, neither the passage of time nor changes in ICU care should have influenced the results presented nor adversely altered the fidelity of the stored airway signals.

To provide for a balanced assessment of the data, analysis was performed in aggregate form and also individually for each patient in the study. Whereas aggregate analysis biased the results in favor of patients with many analyzed epochs, individual analysis amplified the effect of patients with fewer epochs. Regardless of comparison strategy, however, both methods produced nearly identical *C*_rs_ and *R*_rs_ values with negligible bias and exceedingly small LOA.

Although method bias was minimal for both *C*_rs_ and *R*_rs_, the possibility should be acknowledged of introducing a systematic error by the software when calculating the “gold standards” *C*_rs_ and *R*_rs_ by the static method. The cessation of gas flow during the end-inspiratory hold produces a rapid decline in *P*_aw_ from *P*_peak_ to *P*_1_, followed by a slow decay to a plateau *P*_2_ [23]. The timing of the end-inspiratory hold (*t*_hold_) could be an important source of measurement error since a short *t*_hold_ may affect *P*_1_ by the persistence of airflow during inspiratory valve closure or *P*_2_ by prematurely shortening the decay of *P*_aw_. Conversely, a long *t*_hold_ may allow voluntary respiratory muscle activity to occur, also distorting *P*_2_. All breaths in the study were ventilator triggered with no evidence of spontaneous respiratory muscle activity throughout the length of the breath, including the end-inspiratory hold portion. For the cohort, *t*_hold_ was 0.4 [0.4,0.4] (range 0.3 to 0.7) seconds, allowing ample time for inspiratory valve closing [24] and placing *P*_2_ firmly on the flat portion of the plateau, as evidenced by the small decline in *P*_aw_ (< 1.0 cmH_2_O) predicted by decreasing exponentials fitted to the data (*R*^2^ = 0.96) and extrapolated from 0.4 to 1.0 s (Additional file 1: Section S4).

Several assumptions were made in the development of the dynamic method, among them the constancy of *C*_rs_ and *R*_rs_ during insufflation. This basic tenet of the one-dimensional model of Otis et al. [7] is unlikely to hold true during the early stages of inspiration where the volume signal is curvilinear [23]. Past a certain inflation volume, defined here as Δ*V*_LIP_, *C*_rs_ becomes constant and remains so over the rest of the tidal range [25]. The dynamic method was therefore applied to insufflation lung volumes > 200 mL, a Δ*V*_LIP_ chosen to match those reported in ARDS patients [26, 27]. This is probably a conservative estimate since no patient in the study met the Berlin definition for ARDS [28] with half the cohort having normal chest radiographs. Moreover, all patients were ventilated with PEEP_a_ = 5 cmH_2_O, likely resulting in initial lung volumes in the region of constant *C*_rs_. It is possible, however, that small variations in *C*_rs_ and *R*_rs_ during the studied insufflation volumes resulted in the slight uncertainty noted in determining the intersection of the *C*_rs_ – *R*_rs_ functions.

The assumption of absent patient inspiratory effort during insufflation (*P*_mus_ = 0) cannot be independently verified since esophageal balloon catheters were not used in the original study. The validity of this assumption rests on: (1) the use of RRV < 55% as an inclusion criterion, a value noted in heavily sedated ventilated patients [29] and normal individuals during stages of deep sleep; (2) all analyzed breaths were ventilator-initiated; and 3) a cohort of 50 epochs selected randomly from the sample population was characterized by a regular breathing pattern, low respiratory rate (11 [11, 14] bpm) and no signal distortion (see Additional file 1: Section S7 and Table 2e).

The assumption of absent PEEP_i_ also could not be independently verified, but care was taken to include in the analysis only breaths displaying minimal differences between its onset and the prior breath’s end-exhalation *F*_aw_ and *P*_aw_. In addition, (1) no patient in the study was diagnosed with obstructive lung disease; (2) the exhalation time for the cohort allowed ample time for expiration (3.2 ± 0.7 s); and (3) tachypnea (RR > 20 bpm) was absent in all chosen epochs.

The dynamic method is unlikely to perform well under conditions of persistent asynchronous breathing or in the presence of significant respiratory muscle effort. It is also not amenable for bedside use or with ventilators lacking airway signal sampling. Conversely, when used in conjunction with a computer connected to the ventilator’s data-port, the dynamic method may provide accurate ongoing measurements of *C*_rs_ and *R*_rs_ under most clinical conditions encountered during the provision of volume-controlled mechanical ventilation.

Although the present study was not intended as a methodological comparison, the dynamic method appears to perform as well or better than either the LSF or the τ_e_ methods (Additional file 1: Table S3). Unlike these empirical models, the dynamic method represents a deterministic approach to the solution of the equation of motion. As such, it may be applicable to ventilatory modes other than VC and provide insight into the relationship of respiratory system mechanics to other ventilatory variables, such as plateau pressure, respiratory muscle effort and intrinsic PEEP. These, and other issues related to the application of the dynamic method await further confirmation by prospective studies.

### Take-home message

A novel numerical method to calculate static compliance and airway resistance of the respiratory system during ventilatory support is developed and validated.

## Supplementary Information

**Additional file 1: Table S1.** Demographic and ICU Admission Data, Diagnoses, and ICU Admission Chest X Rays for Study Patients. **Table S2.** Average Ventilatory Parameters Computed from All Epochs Used in Method Comparison. **Table S3.** Comparison of bias ± Limits of Agreement (LOA) for C_rs_ and R_rs_ calculated From Individual Patient Data by the Dynamic, Least Square Fitting (LSF) and Expiratory Time Constant (τ_E_) Methods.

**Additional file 2.** Fitting double exponential.

**Additional file 3.** Raw data individual and aggregate.

**Additional file 4.** Experimental data results.

## Data Availability

The datasets used and analyzed during the current study can be found in the Electronic Data Repository. The database storing the raw data is available from the author upon reasonable request.

## Abbreviations

*C*_rs_: Respiratory system static compliance
Δ*V*: Lung volume change during insufflation
Δ*V*_LIP_: Lung volume at the lower inflection point
Δ*V*_UIP_: Lung volume at the upper inflection point
DC: Flow spectrum zero frequency component amplitude
*F*_aw_: Airway flow
FFT: Fast Fourier transform algorithm
H1: First harmonic amplitude of the flow or pressure signal spectrum
*I*: Inertia of the respiratory system
LIP: Lower inflection point
LOA: 95% Limits of agreement
LSF: Multiple least squares fit
*P*_aw_: Airway pressure
PEEP_a_: Applied positive end expiratory pressure
PEEP_i_: Intrinsic PEEP present at end expiration
*P*_mus_: Respiratory muscles applied pressure
*P*_peak_: Peak inspiratory pressure
*P*_plateau_: Plateau pressure during the end-inspiratory hold
*P*_vent_: Ventilator applied pressure
rs: Respiratory system
*R*_rs_: Respiratory system airway resistance
RRV: Respiratory rate variability
τe: Expiratory time constant
*T*_hold_: Timing of the expiratory hold
*t*_k_: A point in time during insufflation
UIP: Upper inflection point
VC: Volume controlled
*V*(*t*): Lung volume as a function of time
*V*_tidal_: Tidal volume
