# Supplementary material for: A novel method to calculate compliance and airway resistance in ventilated patients
Source: Intensive Care Med Exp. 2022 Dec 30;10:55. doi: 10.1186/s40635-022-00483-2 (PMC9800666; doi:10.1186/s40635-022-00483-2)
Supplement: Supplementary file 1 — Additional file 1: Table S1. Demographic and ICU Admission Data, Diagnoses, and ICU Admission Chest X Rays for Study Patients. Table S2. Average Ventilatory Parameters Computed from All Epochs Used in Method Comparison. Table S3. Comparison of bias ± Limits of Agreement (LOA) for Crs and Rrs calculated From Individual Patient Data by the Dynamic, Least Square Fitting (LSF) and Expiratory Time Constant (τE) Methods. [file 40635_2022_483_MOESM1_ESM.docx]

**Electronic Data Repository**

**A Novel Method to Calculate Compliance and Airway Resistance During Mechanical Ventilation**

**Guillermo Gutierrez, MD, PhD**

**Professor Emeritus Medicine, Anesthesiology and Engineering**

**The George Washington University**

**Washington, DC**

**Correspondence:**

Guillermo Gutierrez, MD, PhD

700 New Hampshire Ave, NW

Suite 510

Washington, DC 20037

[gutier@gwu.edu](mailto:gutier@gwu.edu)

**1e. Database description.**

The database used in the present study was collected between March 2011 and February 2012 as part of research on respiratory rate variability (Gutierrez et al, Intensive Care Med. 2013; 39:1359–1367). That research was approved by The George Washington University IRB (IRB No. 110910) that allowed use of the deidentified data in further studies.

The database contains information from 176 patients on positive pressure invasive ventilation with acute respiratory failure of various kinds enrolled within 24 hours of intubation and monitored during the entirety of their ventilatory support. In addition to deidentified demographic information, the database contains flow and pressure signals sampled continuously at 0.032 second intervals (32.25 Hz) from the data-port of the mechanical ventilator (Servo I or Servo S ventilators, Getinge, Solna, Sweden), and saved as contiguous 131.1 seconds long epochs encompassing 4096 samples of each signal.

The database contains approximately half-million epochs stored according to study number. Each patient folder is divided into several folders, each containing specific information, such as the flow and pressure signals, ventilator settings, etc. Each of these folders in turn contains files where data have been saved as 131.1 second longs epochs, with one file for every 24 hours of monitoring.

The dataset is available from the author upon reasonable request.


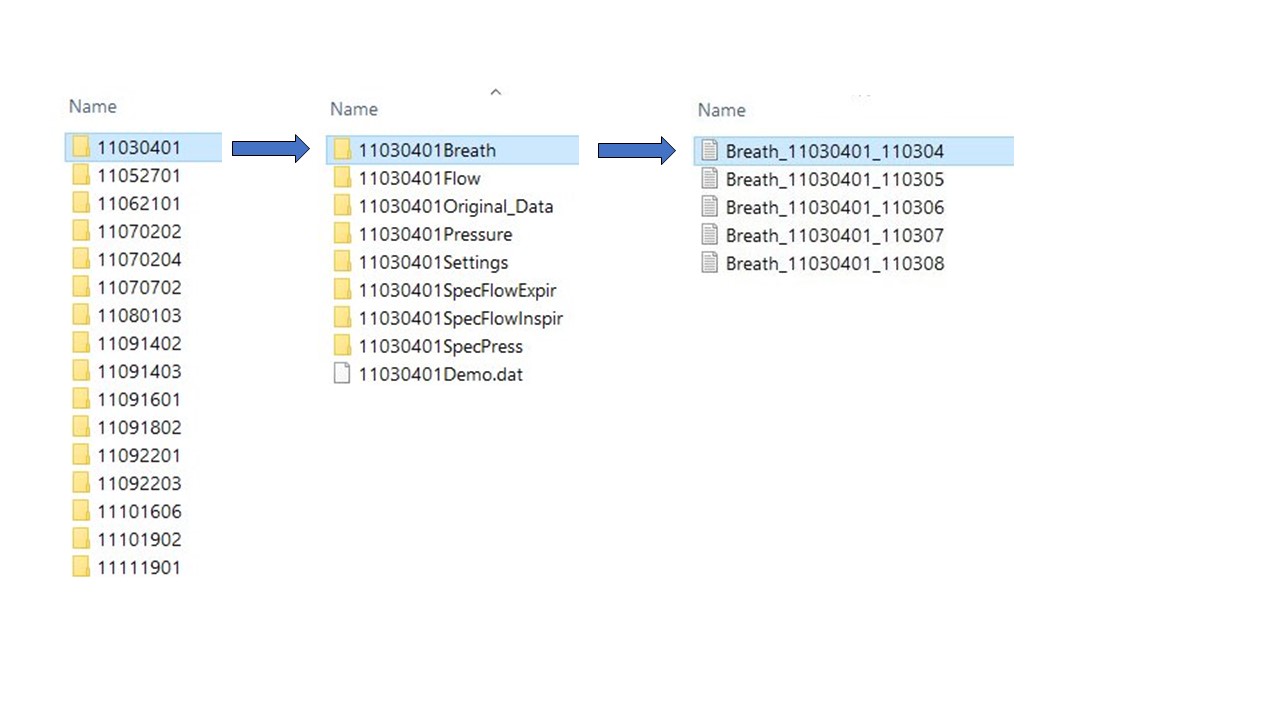


For example, “Breath” files hold data derived from the flow and pressure signals. The first line in a Breath file contains demographic information. Subsequent lines hold values for different calculated variables, such as respiratory rate variability (lines 3,4 and 5 for the inspired flow, expired flow and pressure signals, respectively); FIO_2_ (line 7); respiratory rate (line 8); tidal volumes (line 9), mean pressure (line 10); peak pressures (line 11), etc. Each column contains the average values pertaining to a single epoch. There are as many columns as epochs recorded in a 24-hour period, usually in the neighborhood of 570 epochs.


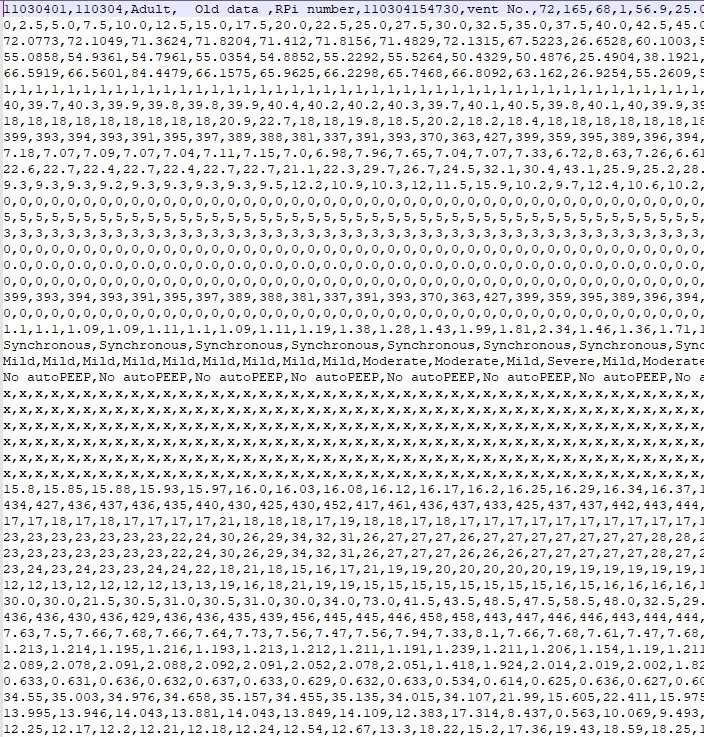


The Flow files contain the sampled flow data.


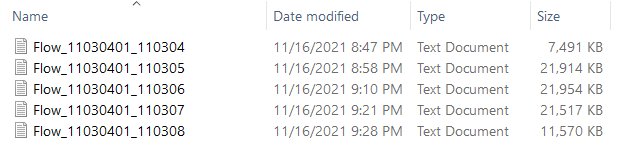


Col 1 = Study number, Col 3 = Epoch time, 4 = Date and time data acquired.

The flow signal (4096 points sampled at 32.25 Hz) begins in column 5.


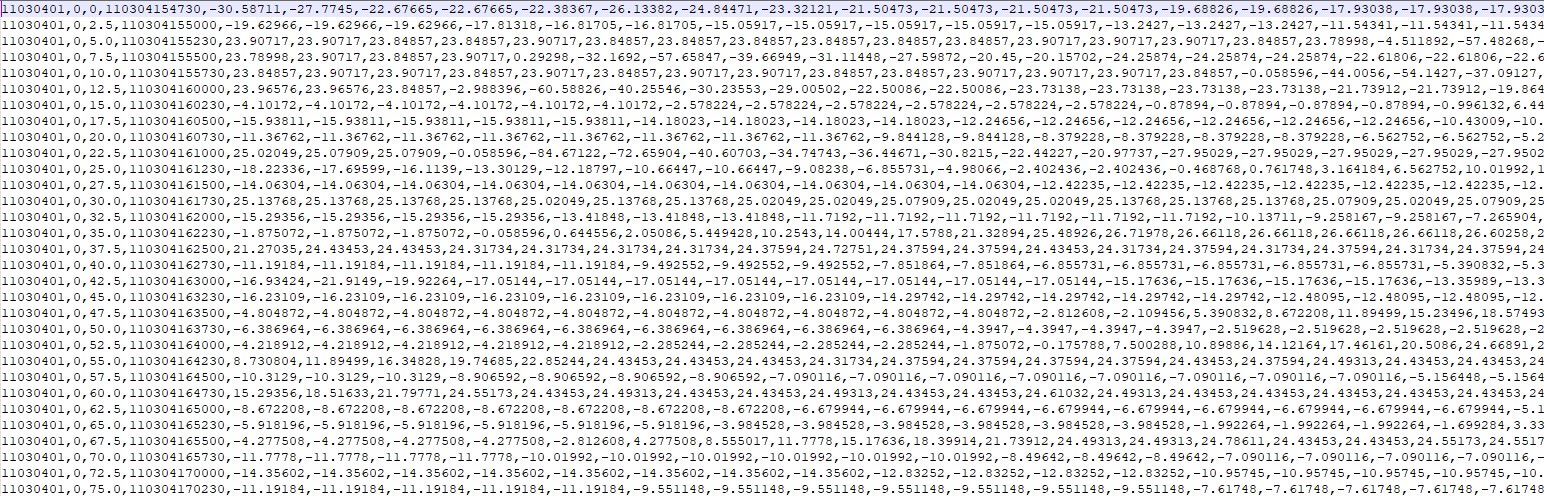


Similarly, the Press files contain the sampled airway pressure data.


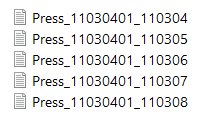


Each line represents one epoch and encompasses 4096 points sampled every 32 ms.


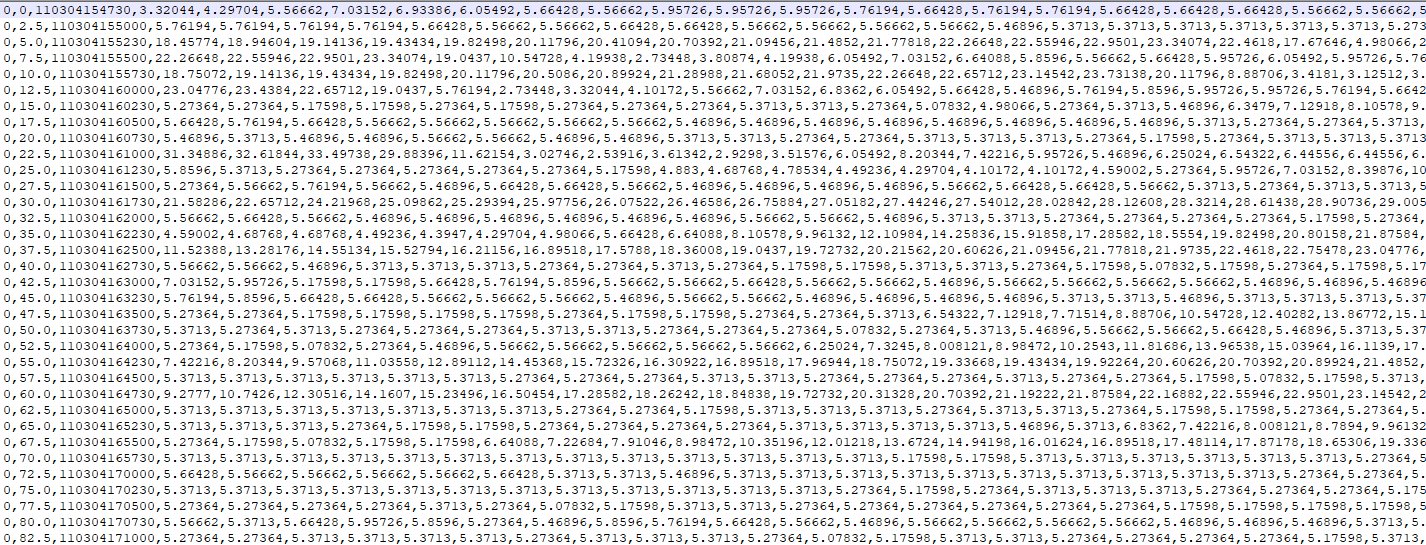


These data are shown graphically in Figure 1e for airway flow and pressure signals stored in a single epoch:

**Figure 1e**

**2e. Example of epoch and individual breath inclusion in the study**.


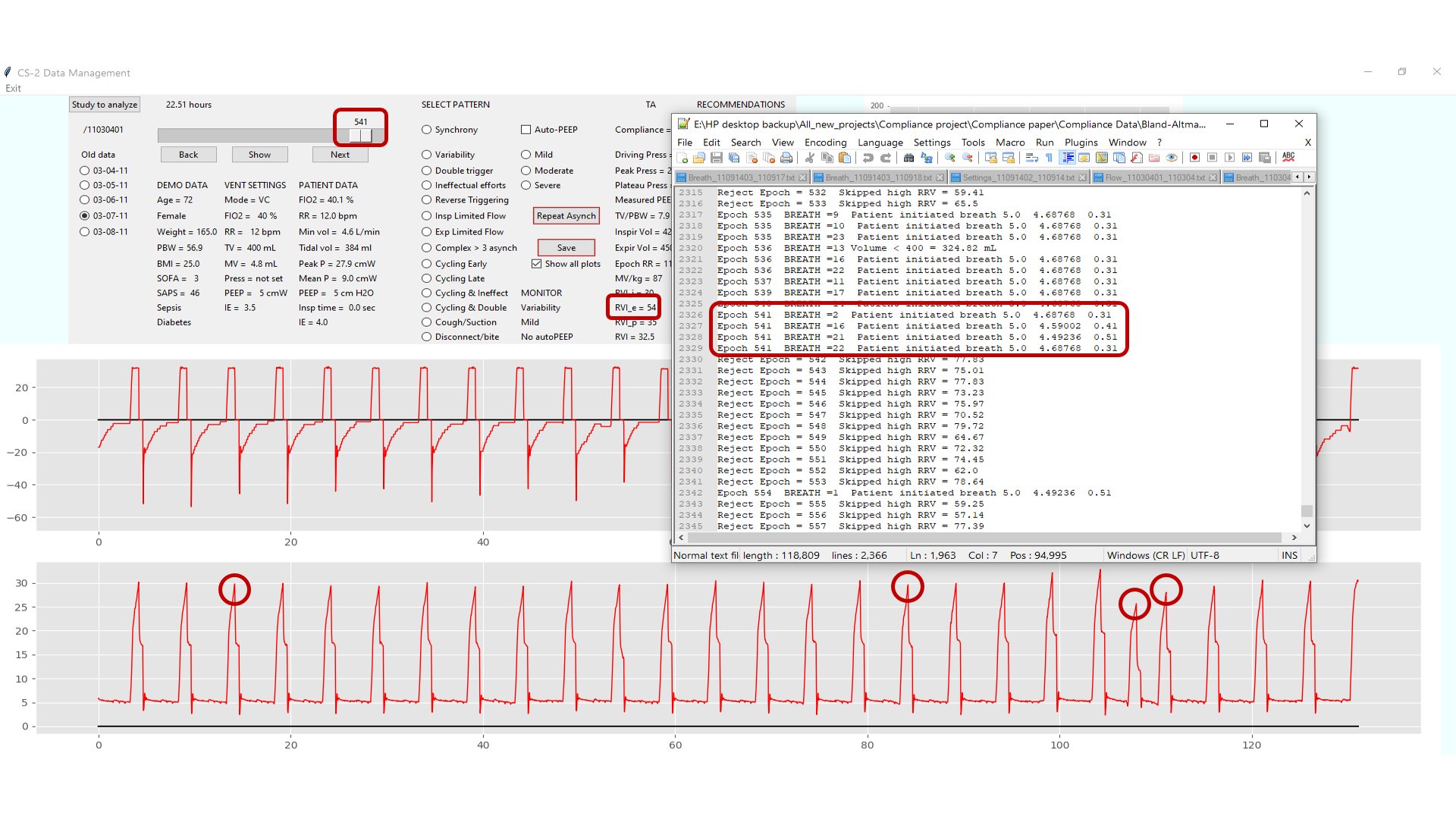
Epoch #541 was acquired at 22:51 hours of the fifth monitoring day, March 7, 2011. The epoch was included in the analysis since RVI ≤ 55% (in this case 54%). The first breath is always rejected to avoid analysis of an incomplete breath. Breaths 2, 16, 21 and 22 were rejected (inset window and red circles) based on possible patient-initiation

Breath 1 2 3 4 5 6 7 8 9 10 11 12 13 14 15 16 17 18 19 20 21 22 23 24

**Figure 2e**

**3e. Numerical application of the method to calculate C_rs_ and R_rs_.**

Software (Python 3.8) was written to identify epochs in the database on volume-controlled ventilation and RRV < 55%. The solution is based on the equation of motion of the respiratory system^[[1]](#footnote-1)^ :

$P_{aw}\left( t \right)= P_{vent}\left( t \right)= \frac{\Delta V\left( t \right)}{C_{rs}}+ R_{rs} F_{aw}\left( t \right)+ {PEEP}_{a}$ (1e)

Once an epoch is identified, the software calculates C_rs_ and R_rs_ by both static and dynamic methods on all breaths meeting selection criteria. Mean epoch values for C_rs_ and R_rs_ are reported.

For the static method, the software calculates C_rs_ = V_tidal_ /(P_plateau_ - PEEP_a_), where V_tidal_ = tidal volume, P_plateau_ = breath-hold P_aw_, and PEEP_a_ = applied positive end expiratory pressure. Similarly, R_rs_ = (P_peak_ - P_plateau_)/F_aw_, where P_peak_ = peak inspiratory pressure and F_aw_ is airway flow measured just prior to breath-holding.

$$solution matrix=\left[ \begin{matrix} P_{aw}\left( R_{1,}C_{1} \right) P_{aw}(R_{2},C_{1}) & \cdots& P_{aw}\left( R_{n}{,C}_{1} \right) \\ \vdots& \vdots& \vdots\\ P_{aw}\left( R_{1},C_{n} \right) P_{aw}(R_{2},C_{n}) & \cdots& P_{aw}\left( R_{n}{,C}_{n} \right) \end{matrix} \right] \begin{matrix} C_{1} \\ \vdots\\ C_{n} \end{matrix}$$

For the dynamic method, solution matrices are generated for each set of F_aw_(t_k_), ΔV(t_k_) and PEEP_a_ measurements obtained at 0.032 second intervals during insufflation from ΔV(t) ≥ ΔV(t)_open_ = 200 mL ^[[2]](#footnote-2),^ ^[[3]](#footnote-3)^ to end-inspiration (t_end-insp_) with the latter identified by a decrease in F_aw_ > 5 L·min^-1^. P_aw_(t) solution matrices are developed by sequentially applying a range of C_rs_ values from 0.05 to 200 mL·cmH_2_O ^-1^ at intervals of 0.05 mL·cmH_2_O ^-1^ and R_rs_ of 0.005 to 50.0 cmH_2_O·s·L^-1^ at intervals of 0.005 cmH_2_O·s·L^-1^ to Eqt. 1e.

A C_rs_-R_rs_ function is then calculated for each P_aw_(t) solution matrix developed from t_open_ to t_end-insp_ by identifying values for C_rs_ and R_rs_ where the solution matrix P_aw_(t) = measured P_aw_(t_k_). The mean (μC_rs_) and standard deviation (σC_rs_) of all generated C_rs_-R_rs_ functions are determined and R_rs_ for the breath is identified by the position along the R_rs_ axis corresponding to the smallest σC_rs_ whereas dynamic C_rs_ = μC_rs_.

**4e.Fitting double exponential.**

**Excel notebook file: Fitting double exponential 4e.xls**

**Excel spreadsheet containing the airway pressure plateau region for the 15 study patients. Also shown are the results of a double exponential y = Ae(-mt) + Be(-nt) fitted to the mean plateau Paw data. Extrapolating the plateau region from 0.4 to 1.0 seconds predicts a decrease in Paw of only 0.8 cmH_2_O (from 17.8 to 17.0 cmH_2_O).**

**
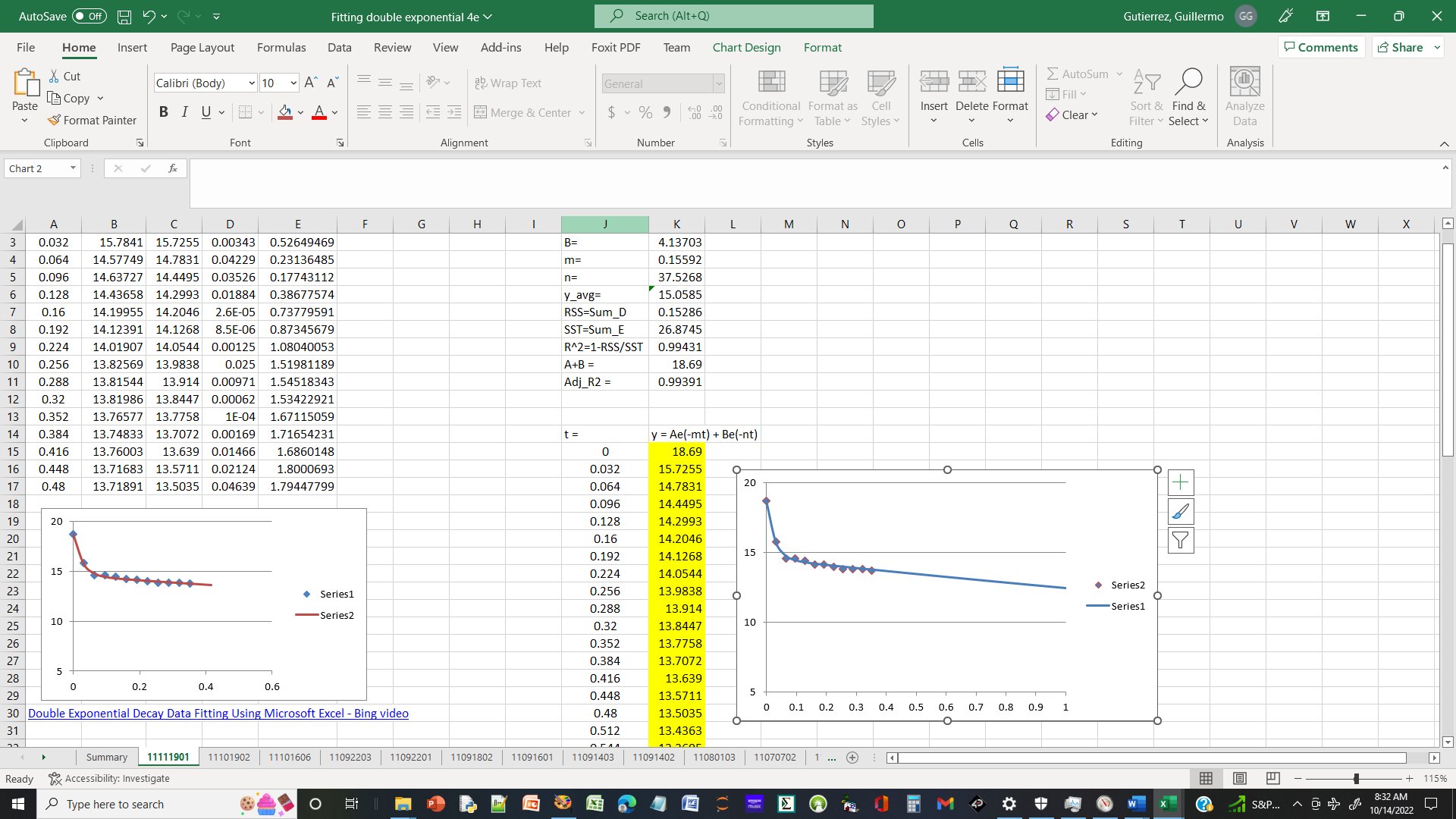
**

Although dynamic method bias was minimal for both C_rs_ and R_rs_, the possibility of introducing an error when calculating the “gold standards” C_rs_ and R_rs_ by the static method should be acknowledged. The cessation of gas flow during the end-inspiratory hold produces a rapid decline in P_aw_ from P_peak_ to P_1_, followed by a slow decay to a plateau P_2_ ^[[4]](#footnote-4)^. The timing of the (t_hold_) is an important source of error since a short t_hold_ may affect P_1_ by the persistence of airflow during inspiratory valve closure or it may prematurely shorten the decay of P_aw_ to P_2_. Conversely, an extended t_hold_ may allow for voluntary respiratory muscle activity to occur, also distorting P_2_ . All analyzed breaths in the study were ventilator triggered with no evidence of respiratory muscle activity, including the time spent in the end-inspiratory hold. t_hold_ for the cohort was 0.4 [0.4,0.4](range 0.3 to 0.7) seconds (Table 1e, Online Supplementary Information). This t_hold_ allowed ample time for inspiratory valve closing^[[5]](#footnote-5)^ and placed P_2_ firmly on the flat portion of the plateau, as evidenced by the small decline in P_aw_ (< 1.0 cmH_2_O) predicted by decreasing exponentials fitted to the data (R^2^ = 0.96) and extrapolated from 0.4 to 1.0 seconds.

**5e. Raw data individual and aggregate.**

**Excel notebook: Raw data - Individual and aggregate 5e.xls**

Contains the results derived from the 3174 chosen epochs.


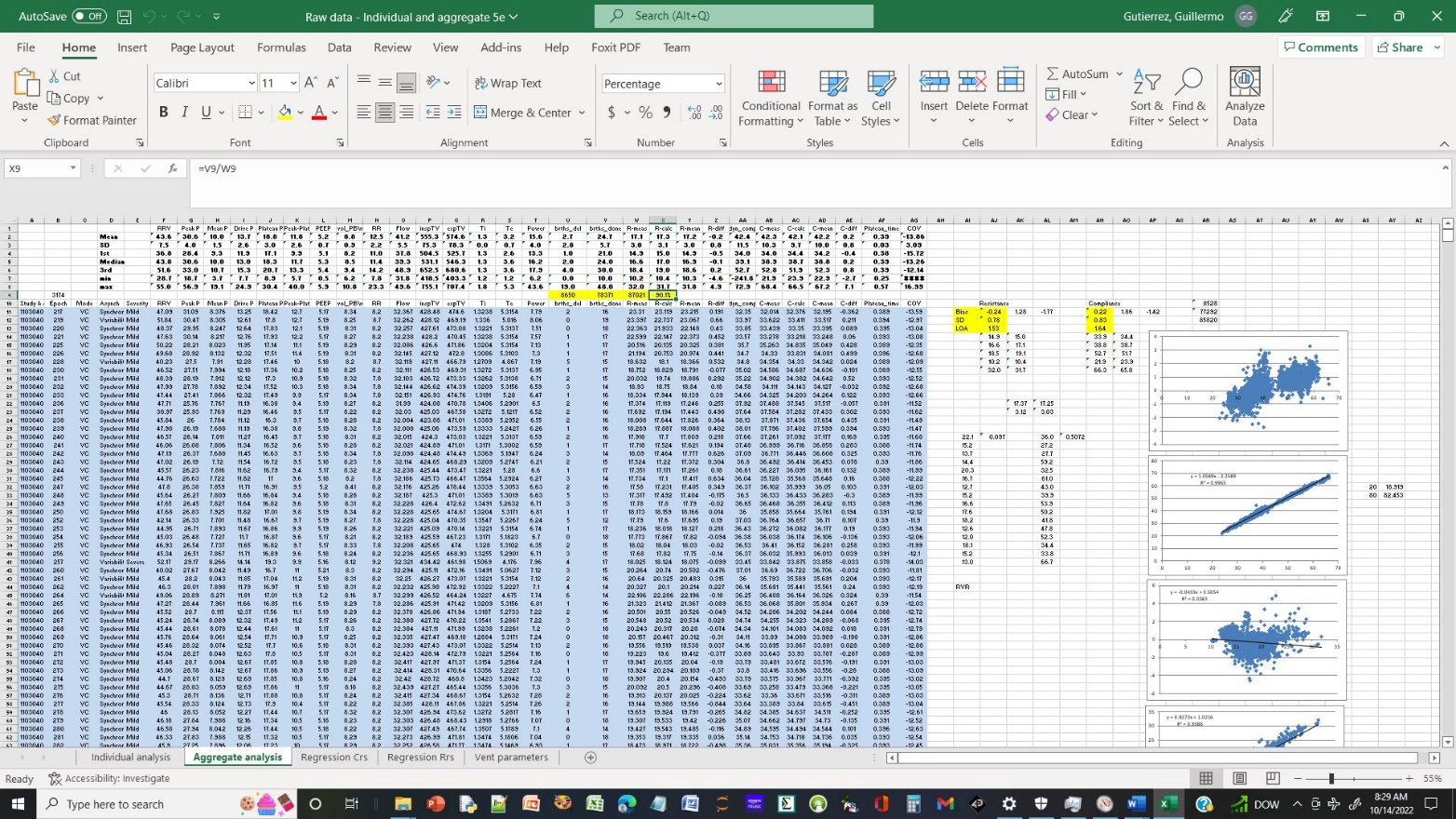


**6e. Experimental data results.**

**Excel notebook: Experimental data with test lung 6e.xls**

Contains data obtained from the 1-liter test lung ventilated with tidal volumes of 300 and 400 mL and three levels of applied PEEP, 0, 5 and 10 cmH2O.

**
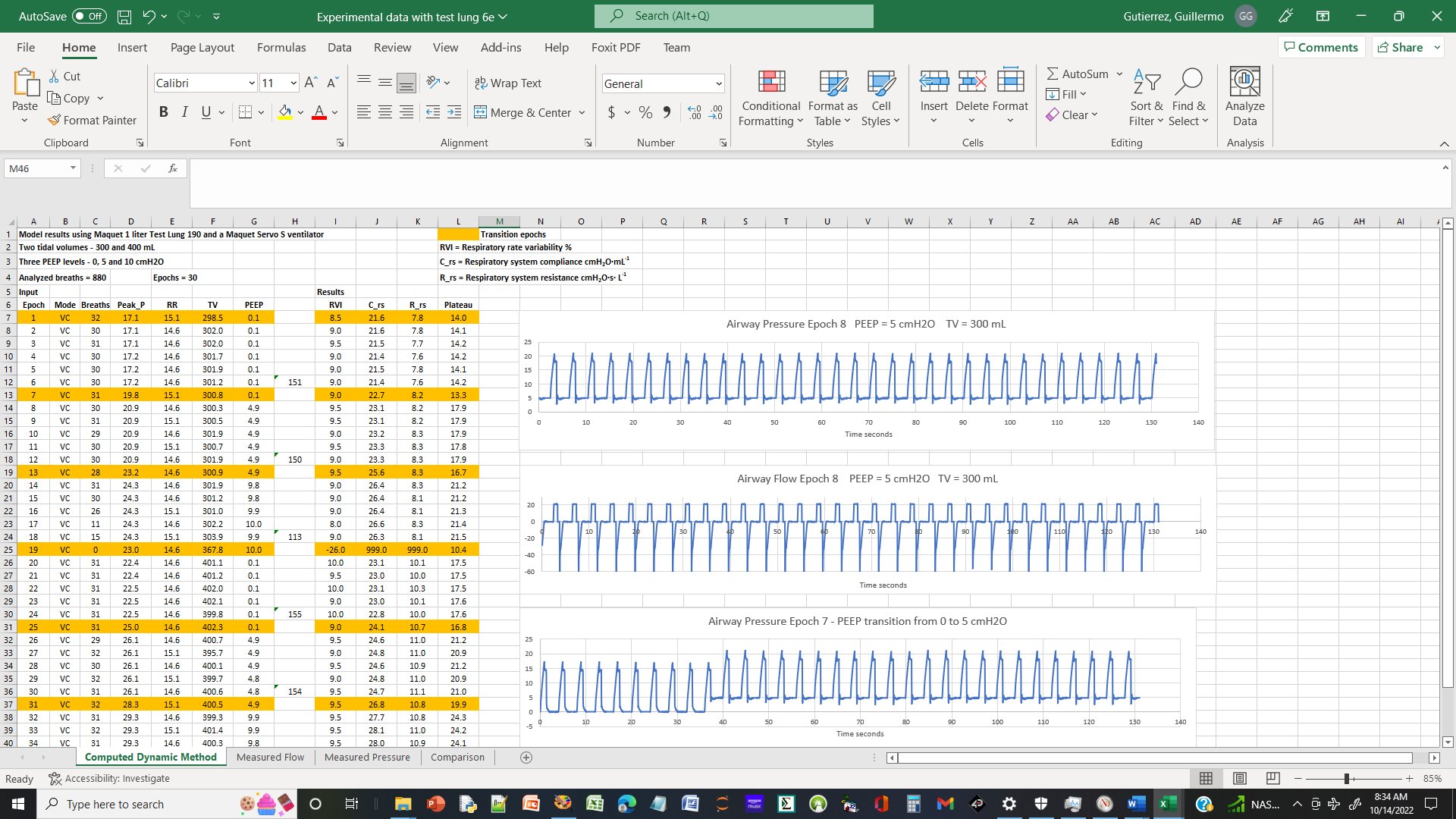
**

**7e. Fifty Randomly Chosen Epochs from a Database of 3174 Epochs (example).**

The criteria used to select the epochs and individual breaths used in the study resulted in epochs displaying regular, steady breathing patterns with low respiratory rates. To further explore this issue, 50 epochs were drawn randomly among the 3171 analyzed epochs (Section 1e, Online Supplementary Information). All breaths within these epochs were uniform in shape, ventilator triggered and displayed no asynchronies, including reverse triggering ^[[6]](#footnote-6)^, findings lending support to the assumption of absent voluntary respiratory muscle activity in the analyzed breaths.


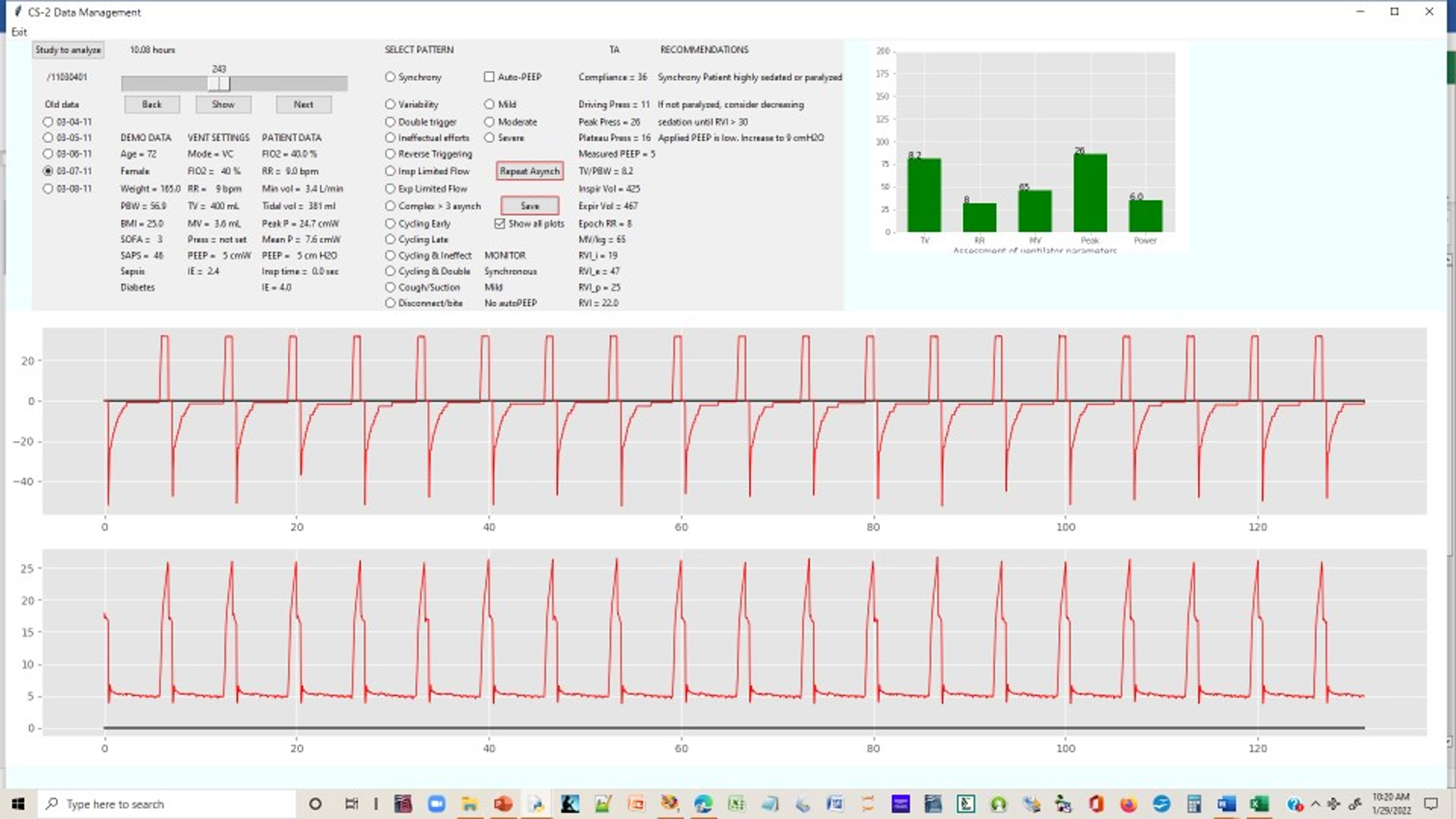


**Table 1e**

**Demographic and ICU Admission Data, Diagnoses, and ICU Admission Chest X Rays for Study Patients.**

| **Patient** | **Gender** | **Age** | **PBW** | **BMI** | **SAPS II** | **P/F** | **Diagnosis** | **Chest X-ray** |
| --- | --- | --- | --- | --- | --- | --- | --- | --- |
| _______________________________________________________________________________________________ | | | | | | | | |
| **1** | F | 72 | 57 | 25 | 46 | 370 | Smoke inhalation | Bilateral effusions |
| **2** | F | 81 | 63 | 45 | 49 | 236 | Pneumonia | Rib fractures |
| **3** | M | 22 | 80 | 22 | 45 | 268 | Trauma | Clear |
| **4** | M | 33 | 52 | 20 | 17 | 553 | Post-operative | Atelectasis |
| **5** | M | 30 | 52 | 29 | 38 | 337 | Post-operative | Pulmonary edema |
| **6** | M | 58 | 80 | 35 | 28 | 215 | Post-operative | Clear |
| **7** | F | 54 | 61 | 22 | 43 | 586 | Cardiac arrest | Clear |
| **8** | F | 42 | 73 | 30 | 32 | 383 | Cardiac arrest | Clear |
| **9** | F | 88 | 54 | 19 | 41 | 298 | Intracranial bleed | Clear |
| **10** | M | 31 | 75 | 19 | 36 | 486 | Sepsis | Atelectasis |
| **11** | M | 51 | 73 | 21 | 33 | 224 | Overdose | Clear |
| **12** | F | 56 | 66 | 18 | 34 | 400 | Intracranial bleed | Clear |
| **13** | M | 37 | 71 | 31 | 19 | 335 | Trauma | Bilateral PNA |
| **14** | F | 75 | 66 | 29 | 62 | 275 | Renal failure | LLL PNA |
| **15** | M | 58 | 78 | 22 | 32 | 458 | Post-operative | LLL PNA |
| **Median** |  | **54** | **66** | **22** | **36** | **337** |  |  |
| **IQR** |  | **[35,65]** | **[59,74]** | **[21,30]** | **[32,44]** | **[272,429]** |  |  |

**Table 2e**

**Average Ventilatory Parameters Computed from All Epochs Used in Method Comparison.**

| **Patient** | **Epochs**  **n = 3174** | **FIO2 (%)** | **Respiratory**  **Rate**  **(bpm)** | **Tidal Volume**  **(mL/PBW)** | **Peak**  **Pressure**  **(cmH_2_O)** | **Mean**  **Pressure**  **(cmH_2_O)** | **Plateau**  **Pressure**  **(cmH_2_O)** | **Driving**  **Pressure**  **(cmH_2_O)** | **Flow**  **(L**·**min^-1^)** | **RRV**  **(%)** |
| --- | --- | --- | --- | --- | --- | --- | --- | --- | --- | --- |
| ______________________________________________________________________________________________________ | | | | | | | | | | |
| **1** | 293 | 40 | 10 | 8.0 | 29 | 9 | 17 | 12 | 32 | 45 |
| **2** | 119 | 100 | 19 | 8.5 | 36 | 14 | 25 | 20 | 40 | 45 |
| **3** | 126 | 50 | 11 | 7.8 | 25 | 9 | 15 | 10 | 42 | 36 |
| **4** | 385 | 40 | 11 | 10.4 | 33 | 10 | 21 | 15 | 37 | 40 |
| **5** | 15 | 60 | 14 | 10.1 | 24 | 9 | 14 | 8 | 36 | 46 |
| **6** | 38 | 40 | 13 | 6.8 | 26 | 10 | 18 | 12 | 40 | 45 |
| **7** | 399 | 50 | 14 | 8.5 | 29 | 10 | 19 | 13 | 39 | 40 |
| **8** | 881 | 40 | 11 | 9.4 | 31 | 10 | 17 | 12 | 49 | 52 |
| **9** | 72 | 60 | 11 | 8.9 | 20 | 4 | 10 | 10 | 34 | 51 |
| **10** | 27 | 30 | 11 | 6.3 | 28 | 9 | 17 | 12 | 37 | 50 |
| **11** | 38 | 50 | 14 | 8.1 | 26 | 9 | 17 | 12 | 42 | 41 |
| **12** | 26 | 40 | 10 | 8.6 | 23 | 8 | 15 | 10 | 38 | 41 |
| **13** | 249 | 40 | 14 | 7.5 | 35 | 12 | 22 | 17 | 42 | 33 |
| **14** | 476 | 40 | 14 | 8.2 | 32 | 11 | 21 | 16 | 39 | 39 |
| **15** | 30 | 40 | 10 | 8.1 | 23 | 8 | 14 | 9 | 41 | 45 |
|  |  |  |  |  |  |  |  |  |  |  |
| **Median** | **119** | **40** | **11** | **8.2** | **28** | **9** | **17** | **12** | **37** | **45** |
| **IQR** | **[34,339]** | **[40,50]** | **[11,14]** | **[7.9,8.8]** | **[24,31]** | **[9,10]** | **[15,20]** | **[10,14]** | **[39,41]** | **[40,46]** |
| **Applied PEEP was 5 cmH_2_O for all epochs.**  **FIO_2_ = Fractional Inspired O_2_ concentration; RRV = Respiratory rate variability; IQR = Interquartile range.** | | | | | | | | | | |

**Table 3e**

**Comparison of bias ± Limits of Agreement (LOA) for C_rs_ and R_rs_ calculated From Individual Patient Data by the Dynamic, Least Square Fitting (LSF) and Expiratory Time Constant (τ_E_) Methods .**

| **Method** | **C_rs_ bias ± LOA**  **(cmH_2_O·mL^-1^)** | **R_rs_ bias ± LOA**  **(cmH_2_O·s· L^-1^)** |
| --- | --- | --- |
| Dynamic (n = 15; s = 3174) | 0.6 ± 0.9 | - 0.1 ± 0.8 |
| LSF (n = 1; s = 11) ^[[7]](#footnote-7)^ | 1.5 ± 3.0 | 4.9 ± 10.2 |
| τ_E_ (n = 24; s = 24)^[[8]](#footnote-8)^ | 2.0 ± 4.5 | - 1.3 ± 2.2 |

n = number of patients in the study; s = number of independent samples.

1. Otis, A.B., Fenn, W.O. and Rahn, H. (1950) Mechanics of Breathing in Man. J Appl Physiol 2: 592-607. doi: 10.1152/jappl.1950.2.11.592. PMID: 15436363. [↑](#footnote-ref-1)
2. Mergoni M, Volpi A, Bricchi C, Rossi A. (2001) Lower inflection point and recruitment with PEEP in ventilated patients with acute respiratory failure. J Appl Physiol (1985) 91:441-450.

   doi: 10.1152/jappl.2001.91.1.441. PMID: 11408462. [↑](#footnote-ref-2)
3. Matamis D, Lemaire F, Harf A, Brun-Buisson C, Ansquer JC, Atlan G. (1984) Total respiratory pressure-volume curves in the adult respiratory distress syndrome. Chest 86:58-66. doi:10.1378/chest.86.1.58 [↑](#footnote-ref-3)
4. Bates JH, Baconnier P, Milic-Emili J.(1985) A theoretical analysis of interrupter technique for measuring respiratory mechanics. J Appl Physiol 1988;64:2204-2214.

   doi: 10.1152/jappl.1988.64.5.2204. PMID: 3391919. [↑](#footnote-ref-4)
5. Bates JH, Hunter IW, Sly PD, Okubo S, Filiatrault S, Milic-Emili J. (1987) Effect of valve closure time on the determination of respiratory resistance by flow interruption. Med Biol Eng Comput 25:136-140.

   doi: 10.1007/BF02442841. PMID: 3695616. [↑](#footnote-ref-5)
6. Akoumianaki E, Lyazidi A, Rey N, Matamis D, Perez-Martinez N, Giraud R, Mancebo J, Brochard L, Richard JCM. (2013) Mechanical ventilation-induced reverse-triggered breaths: a frequently unrecognized form of neuromechanical coupling. Chest 143:927–938. doi:10.1378/chest.12-1817 [↑](#footnote-ref-6)
7. Iotti GA, Braschi A, Brunner JX, Smits T, Olivei M, Palo A, Veronesi R. (1995) Respiratory mechanics by least squares fitting in mechanically ventilated patients: applications during paralysis and during pressure support ventilation. Intensive Care Med. 21:406-413.

   doi: 10.1007/BF01707409. PMID: 7665750. [↑](#footnote-ref-7)
8. Al-Rawas N, Banner MJ, Euliano NR, Tams CG, Brown J, Martin AD, Gabrielli A. (2013) Expiratory time constant for determinations of plateau pressure, respiratory system compliance, and total resistance. Crit Care 17:R23.

   doi: 10.1186/cc12500. PMID: 23384402; PMCID: PMC4056774. [↑](#footnote-ref-8)
